# Adaptation of ventilation strategies from acute RDS to severe BPD: A national multicenter survey of practices in extremely preterm infants

**DOI:** 10.1097/MD.0000000000041973

**Published:** 2025-05-30

**Authors:** Can Akyildiz, Funda Tüzün, Nuray Duman, Abdullah Bariş Akcan, Zeynep Alp Ünkar, Canan Aygün, Şenol Bozdağ, Özlem Bozkurt, Özgül Bulut, Ali Bülbül, Melek Büyükeren, Gökhan Büyükkale, Yalçin Çelik, İstemi Han Çelik, Hasan Çetin, Merih Çetinkaya, Dilek Çoban, Tuğba Egeli, Zeynel Gökmen, Özkan İlhan, Fatih İşleyen, Şebnem Kader, Hasan Kahveci, Gözde Kanmaz, Leyla Karadeniz, Belma Saygili Karagöl, Nejat Narli, Emel Okulu, Hakan Ongun, Mustafa Özdemir, Özmert M.A. Özdemir, Ahmet Özdemir, Hilal Özkan, Hüseyin Şimşek, Sema Tanriverdi, Nuriye Tarakçi, Kadir Şerafettin Tekgündüz, Demet Terek, Özgün Uygur, İpek Güney Varal, Tülin Gökmen Yildirim, Hasan Özkan

**Affiliations:** a Department of Pediatrics, Division of Neonatology, Faculty of Medicine, Dokuz Eylül University, Izmir, Turkey; b Department of Pediatrics, Division of Neonatology, Faculty of Medicine, Adnan Menderes University Medical School, Aydin, Turkey; c Department of Pediatrics, Division of Neonatology, Faculty of Medicine, Istanbul University-Cerrahpaşa, Istanbul, Turkey; d Department of Pediatrics, Division of Neonatology, Faculty of Medicine, Ondokuz Mayis University, Samsun, Turkey; e Department of Pediatrics, Division of Neonatology, Faculty of Medicine, İstanbul Okan University, Istanbul, Turkey; f Department of Pediatrics, Division of Neonatology, Faculty of Medicine, Kocaeli University, Kocaeli, Turkey; g Department of Pediatrics, Division of Neonatology, Istanbul Medeniyet University Goztepe Training and Research Hospital, Istanbul, Turkey; h Department of Pediatrics, Division of Neonatology, University of Health Sciences Türkiye, Sisli Hamidiye Etfal Training and Research Hospital, Istanbul, Turkey; i Department of Pediatrics, Division of Neonatology, Konya City Hospital, Konya, Turkey; j Department of Pediatrics, Division of Neonatology, Kanuni Sultan Suleyman Training and Research Hospital, Istanbul, Turkey; k Department of Pediatrics, Division of Neonatology, Faculty of Medicine, Mersin University, Mersin, Turkey; l Department of Pediatrics, Division of Neonatology, University of Health Sciences Türkiye, Etlik Zübeyde Hanim Women’s Health Teaching and Research Hospital, Ankara, Turkey; m Department of Pediatrics, Division of Neonatology, Medical Faculty, Suleyman Demirel University, Isparta, Turkey; n Department of Pediatrics, Division of Neonatology, Health Sciences University, Başaksehir. Cam and Sakura City Hospital, Istanbul, Turkey; o Department of Pediatrics, Division of Neonatology, Atatürk Sanatoryum Training and Research Hospital, University of Health Sciences, Ankara, Turkey; p Department of Pediatrics, Division of Neonatology, İzmir City Hospital, Izmir, Turkey; q Department of Pediatrics, Division of Neonatology, Medical Faculty of Baskent University, Konya, Turkey; r Department of Pediatrics, Division of Neonatology, Mugla Sitki Kocman University School of Medicine, Mugla, Turkey; s Department of Pediatrics, Division of Neonatology, Şanliurfa Training and Research Hospital, University of Health Sciences, Şanliurfa, Turkey; t Department of Pediatrics, Division of Neonatology, Kirklareli Training and Research Hospital, University of Health Sciences, Kirklareli, Turkey; u Department of Pediatrics, Division of Neonatology, University of Health Sciences Erzurum Medical Faculty, Erzurum, Turkey; v Department of Pediatrics, Division of Neonatology, Zekai Tahir Burak Maternity Teaching Hospital, Ankara, Turkey; w Department of Pediatrics, Division of Neonatology, Istanbul University, İstanbul, Turkey; x Department of Pediatrics, Division of Neonatology, Gulhane Medicine Faculty, University of Health Sciences, Ankara, Turkey; y Department of Pediatrics, Division of Neonatology, Cukurova University, Adana, Turkey; z Department of Pediatrics, Division of Neonatology, Faculty of Medicine, Ankara University, Ankara, Turkey; aa Department of Pediatrics, Division of Neonatology, Faculty of Medicine, Akdeniz University, Antalya, Turkey; bb Department of Pediatrics, Division of Neonatology, Mardin Training and Research Hospital, University of Health Sciences, Mardin, Turkey; cc Department of Pediatrics, Division of Neonatology, Faculty of Medicine, Pamukkale University, Denizli, Turkey; dd Department of Pediatrics, Division of Neonatology, Faculty of Medicine, Erciyes University, Kayseri, Turkey; ee Department of Pediatrics, Division of Neonatology, Faculty of Medicine, Uludag University, Bursa, Turkey; ff Department of Pediatrics, Division of Neonatology, Mersin Training and Research Hospital, University of Health Sciences, Mersin, Turkey; gg Department of Pediatrics, Division of Neonatology, Faculty of Medicine, Celal Bayar University, Manisa, Turkey; hh Department of Pediatrics, Division of Neonatology, Meram Medical Faculty, Necmettin Erbakan University, Konya, Turkey; ii Department of Pediatrics, Division of Neonatology, Faculty of Medicine, Atatürk University, Erzurum, Turkey; jj Department of Pediatrics, Division of Neonatology, Faculty of Medicine, Ege University, Izmir, Turkey; kk Department of Pediatrics, Division of Neonatology, İzmir Tepecik Training and Research Hospital, University of Health Sciences, Izmir, Turkey; ll Department of Pediatrics, Division of Neonatology, Bursa Yuksek Ihtisas Training and Research Hospital, University of Health Sciences, Bursa, Turkey; mm Department of Pediatrics, Division of Neonatology, H.S.U. Dr. Behcet Uz Children’s Education and Research Hospital, University of Health Sciences, Izmir, Turkey.

**Keywords:** bronchopulmonary dysplasia, extremely premature infant, high frequency ventilation, noninvasive ventilation, positive pressure respiration, respiratory distress syndrome

## Abstract

Advances in diagnostic and therapeutic methods have led to a paradigm shift in the management of bronchopulmonary dysplasia (BPD). The lack of evidence-based data in this area has led to variations in clinical practice. The aim of this study was to identify these differences and compare them with recommendations based on pathophysiology. The study was designed as an observational online survey of neonatologists from level 3 to 4 neonatal intensive care units caring for premature infants at increased risk of BPD and born before 28 weeks’ gestation. Respondents were invited to participate in the study through the portal of the Turkish Neonatal Society. Participants were surveyed online about preferred ventilation modes, settings and clinical management of these patients through each respiratory distress syndrome, evolving BPD and severe BPD phases. A total of 39 centers involved in the study. Pressure-control assist-control volume-guaranteed was the most commonly preferred ventilation mode in respiratory distress syndrome and evolving BPD, while high frequency oscillatory ventilation was most commonly used in severe BPD. The use of synchronized intermittent mandatory ventilation volume-guaranteed pressure support ventilation increased with disease progression. Ventilation settings were found to be changed according to pathophysiological recommendations, but not to the extent recommended. The study shows that early ventilation strategies are predominantly maintained in the later phases of BPD, although there are notable differences between centers.

## 1. Introduction

Bronchopulmonary dysplasia (BPD) is a significant morbidity more commonly seen in premature infants and requires prolonged respiratory support. Although advancements in treatment are expected to reduce BPD incidence, most evidence suggests that the rate of BPD has not declined over time. The rising survival chances for extremely premature infants and prolonged ventilation of the extremely immature lungs have also led to different BPD phenotypes necessitating different approach.^[1,2]^

The clinical presentation of modern BPD is generally milder initially, yet some infants remain dependent on ventilators and experience disease progression through its early, evolving, and established phases.^[3]^ In developing individualized ventilation strategies, it is crucial to ventilate each infant based on their unique characteristics and the pathophysiology of the underlying disease. While the primary goal in preterm infants is to focus on lung-protective strategies in the acute phase of the respiratory distress syndrome (RDS), some infants require chronic ventilation, and the lung-protective strategies effective during the acute phase of RDS do not maintain their validity in the chronic phase.^[4]^ A significant misstep among clinicians is to continue ventilating these infants as in the acute phase from the onset of BPD transition, neglecting the altered lung mechanics according to BPD pathophysiology. Although evidence-based data may be lacking for patients with evolving or established BPD, expert recommendations exist considering the underlying pathophysiology.^[1,2,5]^ The strongest recommendations on this issue have been proposed by the BPD collaborative.^[1,2]^ However, point prevalence studies indicate that even among BPD collaborative centers, practices remain heterogeneous.^[6]^ Despite the understanding of pathophysiology and expert opinions, the adaptation process is noted to be challenging.

In Turkey, the preterm birth rate was reported to be 12.9%, and when categorized by gestational age, 3.4% of preterm infants were born extremely preterm (<28 weeks) and 7.4% were born very preterm (28–31 weeks).^[7]^ This national multicenter survey aimed to investigate the ventilation strategies commonly used by different centers, focusing on how these strategies adapt from the RDS phase to the evolving and established phases of BPD.

## 2. Materials and methods

This study was designed as a multicenter observational descriptive online survey. Neonatologists were notified via the Turkish Neonatology Society’s email portal. Additionally, centers meeting the criteria in all geographical regions of the country were contacted verbally and invited to participate in the study. Volunteer centers who met the inclusion criteria were included in the study, with a maximum of 1 participant per center. Participants were instructed to answer the questions in accordance with their unit’s protocols.

The inclusion criteria for the study were as follows: (i) being a consultant neonatologist representing a level 3 or 4 neonatal intensive care unit; (ii) caring for infants born at <28 weeks of gestation; (iii) monitoring patients at risk of BPD and those progressing to BPD; (iv) volunteering to participate. Participants with incomplete or inaccurate response in the survey form, as well as multiple entries from the same center, were excluded from the study. Informed consent was obtained from the participants online. Participants were contacted via email and phone, and the survey form prepared on surveymonkey.com was administered online.

The survey form, consisting of 42 multiple-choice or open-ended questions, was divided into 2 sections. The first section covered demographic data about the centers, while the second section examined the ventilation strategies preferred during the 3 phases of respiratory disease: RDS, evolving BPD (eBPD), and severe BPD (sBPD). For eBPD, questions addressed infants who remained on mechanical ventilation for more than 4 weeks, whereas for sBPD, they focused on infants still dependent on invasive mechanical ventilation at 36 weeks. The demographic data included characteristics such as the number of level 3 and 4 neonatal intensive care unit (NICU) beds, the annual number of infants born before 28 weeks of gestation, the BPD definition used in the clinic, the most common phenotype of BPD observed, the annual number of BPD cases requiring tracheostomy, the annual mortality rate due to BPD, and the number of patients discharged with home oxygen therapy (Supplementary File 1, Supplemental Digital Content, https://links.lww.com/MD/P12).

Ventilation modes preferred during RDS, eBPD, and sBPD (assist-control [AC], assist-control volume-guaranteed [AC-VG], synchronized intermittent mandatory ventilation [SIMV], synchronized intermittent mandatory ventilation volume-guaranteed [SIMV-VG], high frequency oscillatory ventilation [HFOV], high frequency oscillatory ventilation volume guaranteed, pressure support ventilation [PSV], pressure support ventilation volume guaranteed [PSV-VG], synchronized intermittent mandatory ventilation pressure support ventilation volume-guaranteed [SIMV-PSV-VG], and neurally adjusted ventilatory assist [NAVA]) were assessed using a Likert scale ranging from 1 (least frequently used) to 5 (most frequently used). The most commonly preferred values for each ventilation parameter (positive end expiratory pressure [PEEP], mean airway pressure, peak inspiratory pressure, maximum inspiratory pressure, amplitude, tidal volume [VT], inspiration time, inhalation exhalation ratio [I:E], rate) were separately queried for each of the 3 periods.

The preference for noninvasive ventilation modes (nasal intermittent positive pressure ventilation [NIPPV], continuous positive airway pressure, synchronized nasal intermittent positive pressure ventilation, nasal high frequency oscillatory ventilation, high flow nasal cannula, NAVA) during the transition from invasive mechanical ventilation to noninvasive methods after the extubation of intubated sBPD patients was also assessed using a Likert scale ranging from 1 (least frequently used) to 5 (most frequently used). Bedside monitoring methods, targeted oxygen saturation (SpO_2_) and partial carbon dioxide (pCO_2_) values, sedative preferences, echocardiographic screening time for pulmonary hypertension, and parameters considered when deciding on tracheostomy were also investigated.

The survey responses were accessed through the website, transferred to a statistical program, and analyzed.

The study was approved by the Non-Interventional Research Ethics Committee of Dokuz Eylül University (approval number: 2024/09-11). Informed consent was obtained from all participants in accordance with the Helsinki Declaration.

## 3. Statistical analysis

Statistical analysis was performed using IBM SPSS v.25. Categorical data from the survey were presented as numbers and percentages. Numerical data were assessed for normal distribution. Normally distributed data were presented as mean (±SD); non-normally distributed data were presented as median and interquartile range (IQR). Averages of parameters asked using the Likert scale were displayed with bar graphs, and the frequencies of parameters used in treatment and follow-up were displayed with pie charts. The most frequently used ventilation mode for each center was shown with a heat map. Differences in ventilation parameters across the 3 disease phases were compared using one-way ANOVA.

## 4. Results

The study was completed with the participation of 39 out of 43 centers that met the criteria, ensuring a high level of representation from all regions of Turkey. Responders completed the survey between March 18, 2024 and April 19, 2024. The median number of level 3 to 4 neonatal NICU beds in the participating centers was 25 (IQR 16–36), and the annual number of hospitalized premature infants born before the 28th gestational week was 40 (IQR 30–105).

The frequency of use of the 2001 National Institute of Child Health and Human Development (NICHD), 2018 NICHD, and 2019 Neonatal Research Network BPD classifications in the centers was 35.9%, 33.3%, and 30.8%, respectively. The most prevalent phenotype of BPD was identified as the new BPD, accounting for 56.4% of cases, followed by the combined type (a mixture of new and old BPD) with a frequency of 38.5%. The annual numbers of patients with sBPD, tracheostomy, discharges home with oxygen therapy, and deaths are shown in Table 1.

**Table 1:** Descriptive characteristics of centers.

Feature	n (IQR)
Number of level 3–4 NICU beds	25 (16–36)
Number of babies born at < 28 gestational weeks admitted in approximately 1 yr	40 (30–105)
BPD classification used n (%)	
2001 NICHD	14 (35, 9%)
2018 NICHD	13 (33, 3%)
2019 Neonatal Research Network	12 (30, 8%)
The most common type of BPD in recent years n (%)	
Old BPD	2 (5, 1%)
New BPD	22 (56, 4%)
Old and new BPD together	15 (38, 5%)
Number of patients requiring mechanical ventilation in the 36th postmenstrual week due to severe BPD in the last 1 yr	3 (1–5)
Number of patients requiring tracheostomy due to severe BPD in the last 1 yr	1 (0–1)
Number of patients resulting in mortality due to severe BPD in the last 1 yr	1 (0–1)
Number of patients discharged with home oxygen support in the last 1 yr	1 (0–3)

BPD = bronchopulmonary dysplasia, NICHD = National Institute of Child Health and Human Development, NICU = neonatal intensive care unit.

**Figure 1.**
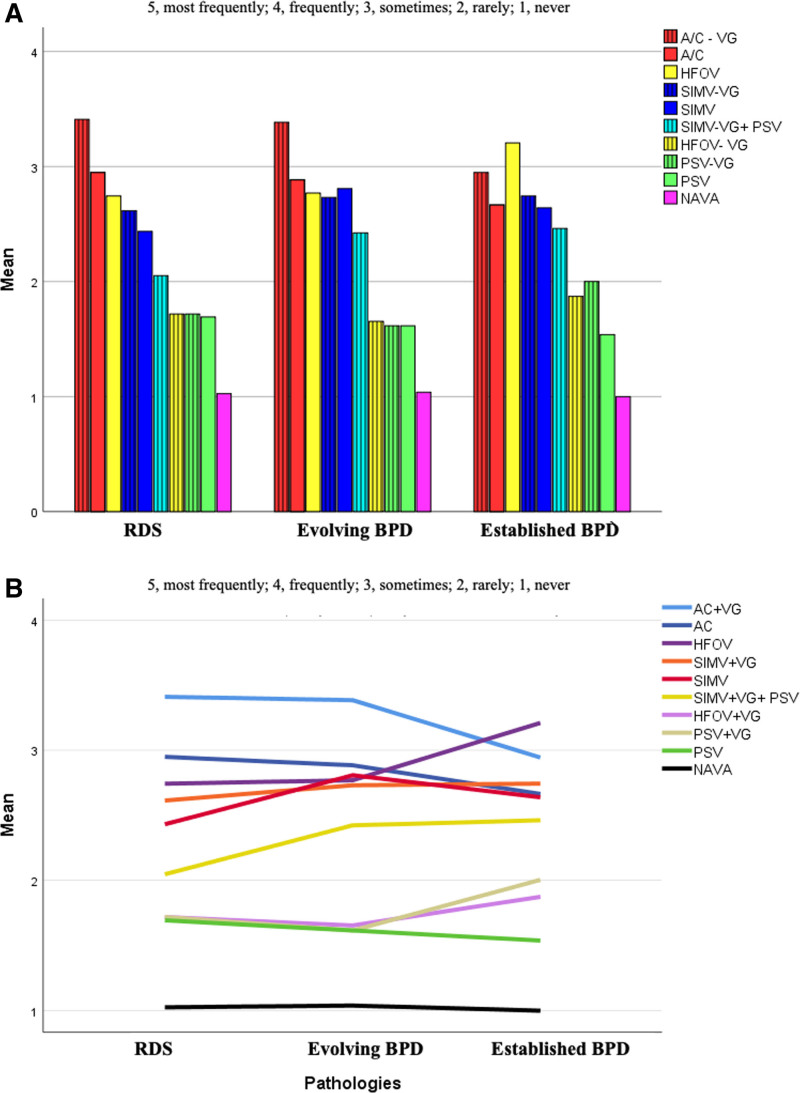
(A) Preferred ventilation modes according to the phases of respiratory disease. (B) Variation in preferred ventilation modes according to the phases of respiratory disease.

The most preferred ventilation mode by the centers during RDS and eBPD periods was AC-VG, whereas the most preferred mode in sBPD was HFOV. The ventilation modes most frequently preferred for all pathologies were AC-VG, AC, HFOV, SIMV, and SIMV-VG (Fig. 1A). It was observed that there was an increase in the preference for HFOV and SIMV-VG-PSV, and a corresponding decrease in the preference for AC or AC-VG, as the patients progressed to the sBPD (Fig. 1B). PSV, PSV-VG, and high frequency oscillatory ventilation volume-guaranteed modes were relatively less preferred in these patients, and NAVA was hardly ever preferred.

**Figure 2.**
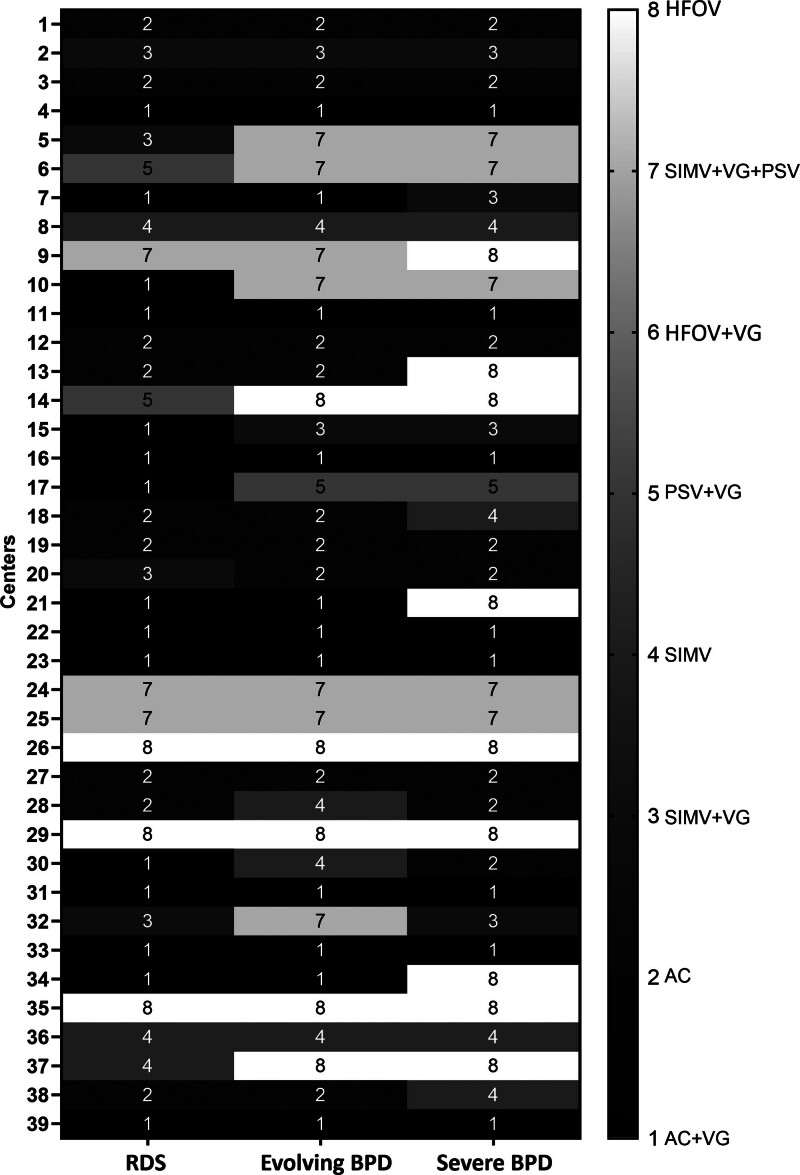
The most preferred mode of mechanical ventilation by the centers according to the phases of respiratory disease.

The heat map demonstrated that approximately 50% of the centers did not change their preferred mode of ventilation during the transition from RDS to sBPD, while the remaining centers exhibited different patterns of change (Fig. 2).

The most commonly preferred mechanical ventilation settings are illustrated for all 3 disease phases. In the progression from RDS to sBPD, it was observed that the most frequently used settings for inspiratory time, PEEP, VT and peak inspiratory pressure, increased, while the respiratory rate decreased. This difference between the groups was statistically significant. Also, in HFOV, statistically significantly higher mean airway pressure and VT were used as the disease progressed, but there was no significant difference in other HFOV parameters (Table 2).

**Table 2:** Most frequently used ventilation settings according to BPD developmental stages.

Feature	RDS	Evolving BPD	Established BPD	*P*
Conventional ventilation				
Tins	0.35 (0.30–0.35)	0.40 (0.35–0.40)	0.45 (0.40–0.50)	**0**
PEEP	6 (5–6)	6 (6–7)	7 (6–8)	**0**
Rate	40 (40–50)	40 (30–40)	30 (20–40)	**0**
i.e.	0.50 (0.33–0.50)	0.37 (0.33–0.50)	0.33 (0.25–0.50)	.16
VT*	5 (5–5.5)	6 (6–6)	7 (6–8.5)	**0**
PIP†	20 (16–22)	25 (19–25)	25 (22–28)	**0**
High frequency ventilation				
Frequency	11 (10–12)	10 (10–10)	9.79 ± 0.58	.08
MAP	10.94 ± 0.54	12.60 ± 0.58	14.00 ± 0.85	**.01**
IE	0.50 (0.50–1.00)	1.0 (0.53–1.00)	1.0 (0.50–1.00)	.1
VT*	1.68 ± 0.12	2.11 ± 0.21	2.29 ± 0.20	**.05**
Amplitude†	21.7 ± 2.5	24.9 ± 3.8	31.3 ± 5.0	.25

Data with normal distribution are shown as mean ± SD, data with no normal distribution are shown as median (IQR). One-way anova was used to compare the data with normal distribution and Kruskal–Wallis analysis was used to compare the data without normal distribution.

IE = inhalation exhalation ratio, IQR = interquartile range, MAP = mean airway pressure, PEEP = positive end expiratory pressure, Tins = inspiration time, VT = tidal volume.

* Represents the setting made in volume-guaranteed modes.

† Represents the setting in modes without volume guarantee.

**Figure 3.**
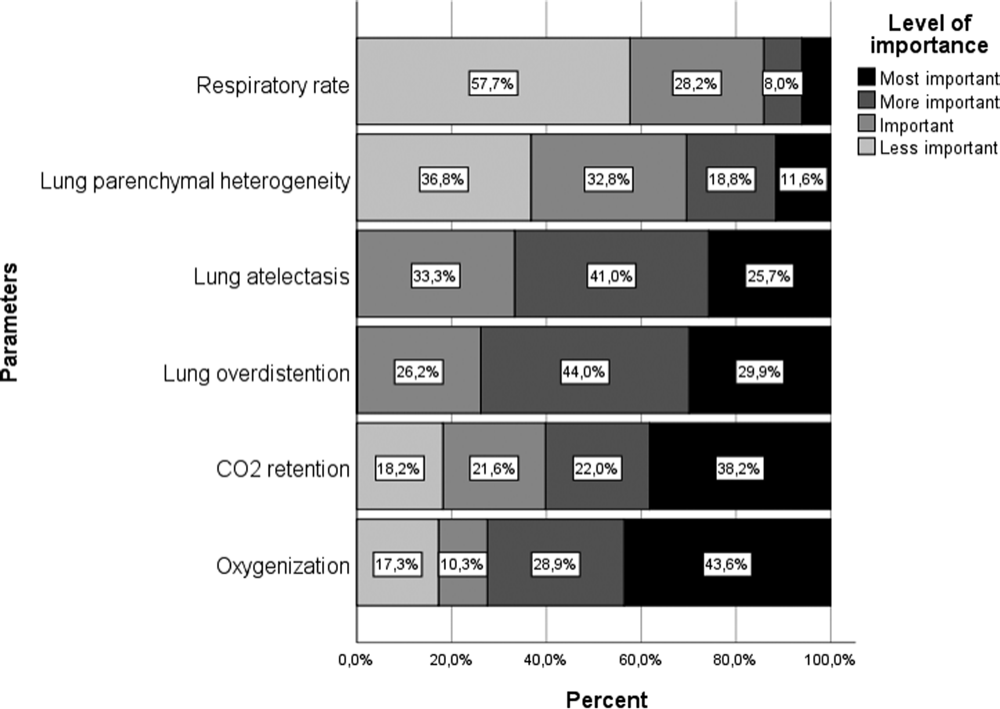
The most considered criteria according to their importance when setting tidal volume, inspiratory time and frequency during ventilation of patients with severe BPD. BPD = bronchopulmonary dysplasia.

The most important parameters considered when deciding on the mechanical ventilator settings of these patients in the centers are oxygenation, carbon dioxide retention, lung overventilation, lung atelectasis, parenchymal heterogeneity, and respiratory rate from the most common to the least common. Parenchymal heterogeneity and respiratory rate were found to be the most common and least important parameters when deciding on the mechanical ventilator settings of these patients in the centers (Fig. 3).

**Figure 4.**
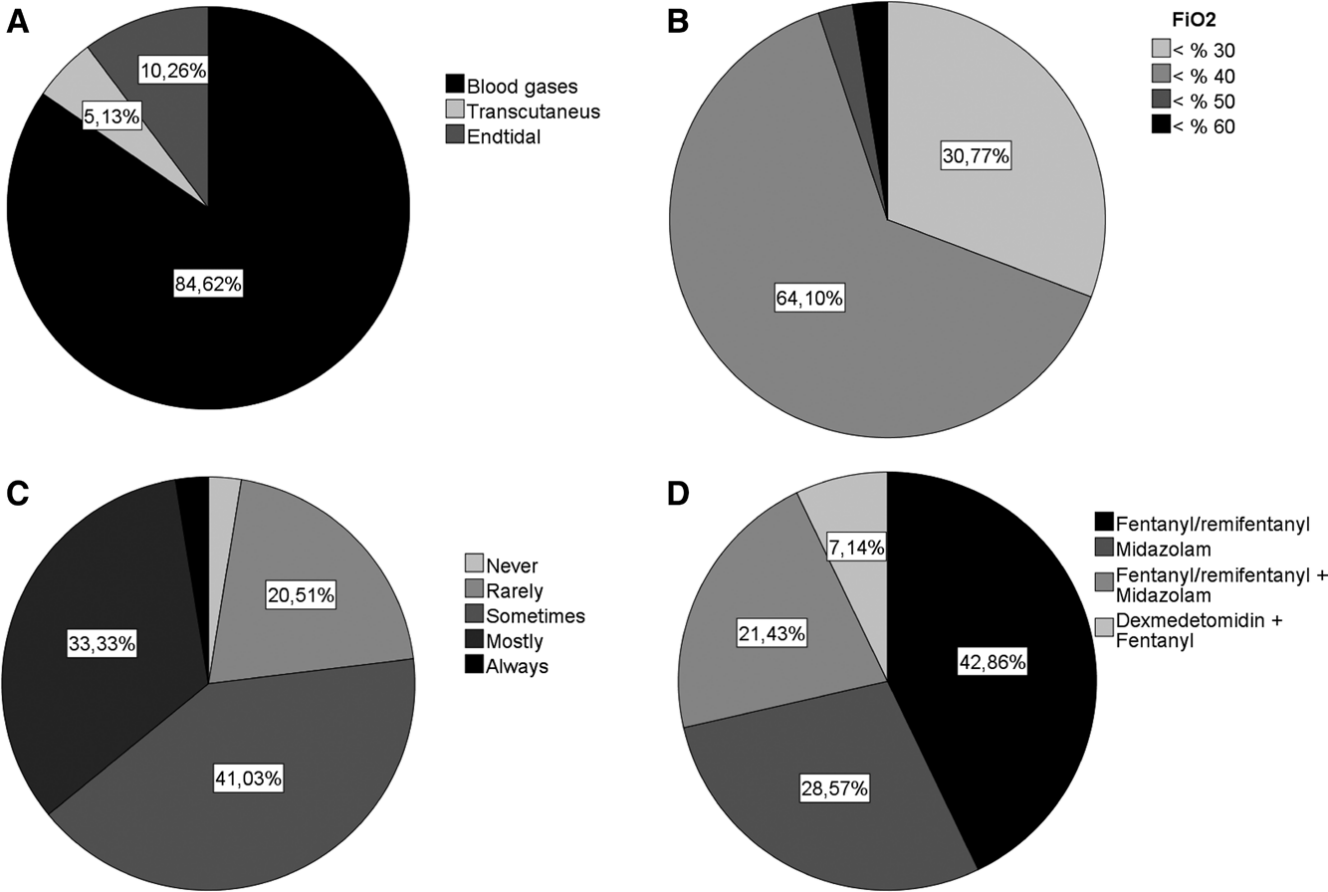
Follow-up of patients with severe BPD. (A) Preferred method of pCO_2_ monitoring. (B) Maximum FiO_2_ value for extubation planning. (C) Frequency of sedative analgesic use. (D) The most preferred drug in sedative analgesia. BPD = bronchopulmonary dysplasia.

Blood gas analysis (84.6%) was the most frequently used method of monitoring pCO_2_ levels in patients with sBPD (Fig. 4A). The mean blood pCO_2_ targets in these patients were 45 to 65 mm Hg (minimum–maximum) and arterial oxygen saturation (SpO2) targets were 90% to 94% (minimum–maximum). The most commonly used fraction of inspired oxygen (FiO_2_) level for extubation planning was 40% (Fig. 4B). The sedation preferences of the centers for sBPD are shown in the graphs (Fig. 4C, D).

**Figure 5.**
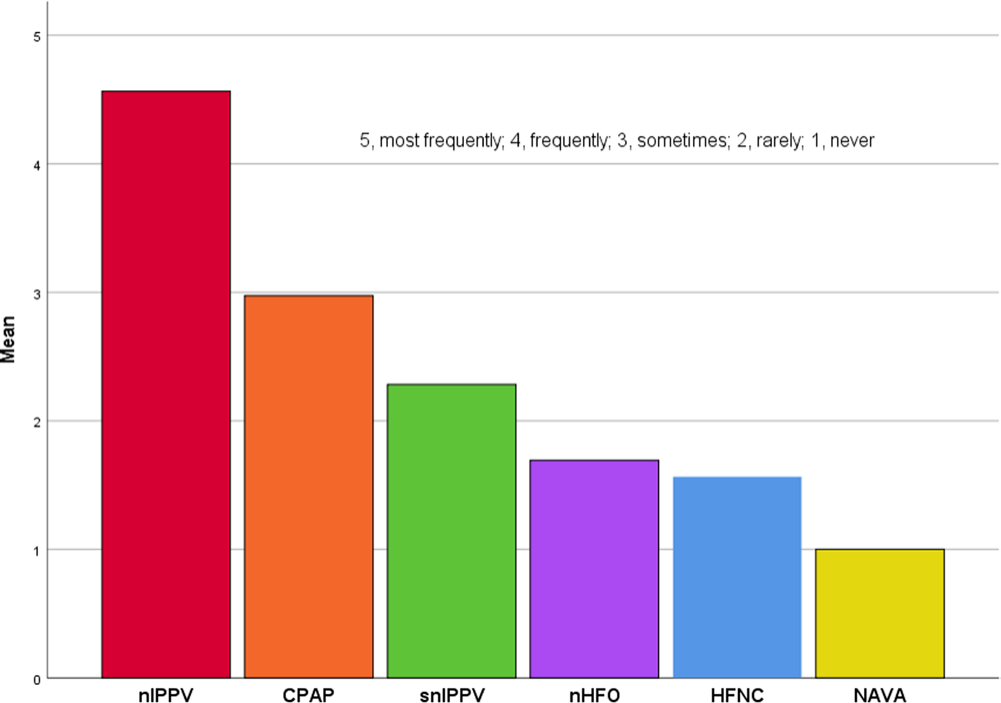
Frequency of noninvasive ventilation mode preference after extubation in patients with severe BPD. BPD = bronchopulmonary dysplasia.

The most preferred mode of noninvasive ventilation following extubation was NIPPV (Fig. 5). The highest tolerated pCO2 level in patients receiving noninvasive ventilation was 65 mm Hg (IQR 65–70), and the highest FiO2 was 60% (IQR 50–60).

Echocardiographic screening for pulmonary hypertension in BPD patients was standard practice in 34 (87.2%) centers. The median gestational age at which this screening was performed was 36 weeks postmenstrual age (PMA) (IQR 34–36). The median number of postnatal systemic steroid courses for BPD was 2 (IQR 2–3).

**Figure 6.**
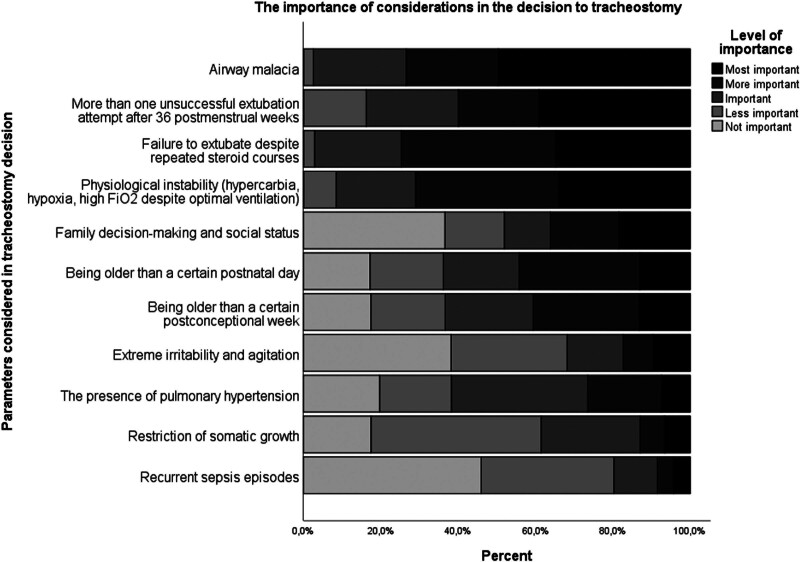
The most considered criteria according to their importance when deciding on tracheostomy in severe BPD. BPD = bronchopulmonary dysplasia.

The most crucial factors considered in the decision to perform a tracheostomy in patients with sBPD were airway malacia, the occurrence of more than 1 unsuccessful extubation attempt after 36 weeks PMA, the inability to extubate despite steroid therapy, and the presence of physiologic instability with hypercarbia, hypoxia and a high FiO2 requirement despite optimal ventilation (Fig. 6). The median timing for considering tracheostomy in infants with severe BPD who are dependent on mechanical ventilation was the 44th postmenstrual week (IQR 36–64). Some centers also considered postnatal days (n = 23), with the median timing being 120 days (IQR 90–150).

## 5. Discussion

This is the first study to investigate in detail the ventilation strategies preferred by different level 3 or 4 NICUs at various stages of the respiratory course in preterm babies, including RDS, evolving BPD, and severe BPD, at a national level. Results reveal that half of the centers did not change their ventilation strategies and oxygenation targets as the patients progressed from RDS to BPD, which is inconsistent with current recommendations, and there were apparent differences among center practices.

The classification of BPD has evolved with the disease since 1967 and various classifications have been proposed and used. The 2001 NICHD classification based on the duration of oxygen therapy for at least 28 days and its concentration in PMA at 36 weeks was updated in 2018 NICHD with the addition of radiological findings and new noninvasive strategies.^[8]^ Lastly, in 2019 Neonatal Research Network study by Jensen, BPD classification was made according to the characteristics of positive pressure support given at 36 weeks PMA.^[9,10]^ Although the 2001 definition is more frequently used, the results show that the use of the newer 2018 NICHD definition and the 2019 Jensen definition is approaching similar rates.

The “new BPD” is characterized by arrested alveolar development, larger alveoli, and reduced surface area for gas exchange, establishing early in evolving phase of BPD within days to weeks. Normally, mechanical strain during inhalation, along with various transcription and growth factors, guides alveolar formation, with elastin and collagen stabilizing the alveolar structure. However, in later stages of BPD, imbalanced deposition and altered cross-linking of elastin and collagen result in radiological and histological features resembling the “old BPD,” with fibrosis and emphysema. These changes create a phenotype in today’s sBPD patients that combines elements of both “new” and “old” BPD.^[2]^ The survey showed that while the most common phenotype is new BPD at 56.4%, combined type BPD is also prevalent at 38.5%.

The acute period of RDS is a period where lung-protective ventilation methods, particularly noninvasive ventilation and volume-guaranteed ventilation techniques, are predominant. Also, in recent years, HFOV has become prominent as a lung-protective alternative in the early period, especially in extremely small preterm infants. The study shows that the preference of volume-guaranteed ventilation in the early period was generally higher in the acute period of RDS, with A/C mode being preferred more than SIMV.

As BPD progresses, the lung becomes more heterogeneous and different from the acute phase. In this evolving phase, radiological findings and lung mechanics lead clinicians to suspect and predict progression to sBPD. Despite the limited evidence on ventilatory strategies for this phase of the disease, the BPD Collaborative recommends starting chronic pulmonary ventilation in infants who have been on mechanical ventilation for at least 4 weeks and/or have reached 32 weeks PMA, especially if they are not adequately responding to standard ventilatory methods.^[2]^ However, clinicians often try to wean support and avoid intermittent positive pressure ventilation during this period leading to atelectasis, worsening ventilation/perfusion mismatch and increased requirements for fraction of inspired oxygen and work of breathing.^[2]^ Our results indicated that in a significant number of centers, the strategies applied during the RDS phase were continued into the evolving BPD phase, without any changes in modes or settings to accommodate the changing lung conditions.

The use of hybrid modes, such as SIMV-PSV-VG, has been proposed as a means of providing optimal ventilation of the various compartments within the established BPD lung, with the additional potential benefit of promoting growth.^[2,11]^ The point prevalence study from BPD collaborative centers indicated that SIMV is a commonly used mode of ventilation in this patient group. However, there was a significant heterogeneity among centers and poor adherence to the collaborative’s recommendations for BPD ventilation.^[6]^ The present survey (our survey) revealed an increase in the use of SIMV-PSV-VG in severe BPD consistent with the recommendations, but use of HFOV is also increased. Approximately half of the centers preferred the same mode at different disease stages (Fig. 2). The increased use of HFOV is thought to stem from attempts to ventilate as if the lung parenchyma were homogeneous, without considering the heterogeneous compliance and resistance seen in advanced BPD. Additionally, it reflects efforts to avoid the high pressures associated with conventional ventilation.

The use of NAVA in patients with sBPD is a promising option, as it has been shown to increase patient-ventilator synchrony, reduce the need for sedation, and improve gas exchange. However, the frequency of use remains low due to the lack of conclusive evidence on this subject.^[12]^

Throughout the chronic stage of BPD, lower ventilation rate (12–20/min), higher tidal volume (10–15 mL/kg), longer inspiratory time (0.5–1 s), lower I:E ratio (0.2) and higher PEEP (7–9 cmH_2_O) are recommended in ventilation settings.^[2]^ Although there were differences in settings between centers, overall the study showed that there were only limited changes to the relevant settings in this stage (Table 2). Most centers do not adhere to these settings, even in severe BPD patients who are dependent on mechanical ventilation. However, when asked in the survey, most centers reported that, in addition to considering carbon dioxide retention and oxygenation, they also take the condition of the lung parenchyma into account when adjusting settings (Fig. 3).

There is a paucity of data in the literature on optimal pCO2 and SPO2 levels and their monitoring in patients with sBPD. The most commonly preferred method for pCO2 in the study was blood gas, with centers targeting a mean range of 45 to 65 mm Hg. In the literature, pCO2 targets vary between 42 and 60 mm Hg.^[13]^ Recommended saturation targets in sBPD vary between 92% and 95%, and in the presence of pulmonary hypertension this target has been suggested to be 95% to 98% in relevant guidelines.^[14]^ In this study, it was observed that most centers set their saturation targets to 90% to 94%, in line with the recommendations for the RDS period (Fig. 4A).

Secondary pulmonary hypertension develops in 25% of cases with moderate to severe BPD due to vascular changes in the lungs. Therefore, screening for pulmonary hypertension by echocardiography at 36 weeks PMA and at discharge is recommended in infants with BPD.^[14]^ Below 28 weeks of gestational age, pulmonary hypertension screening with ECHO is recommended in cases of severe respiratory distress, prolonged oxygen requirements, poor growth and unsatisfactory clinical improvement. In line with the recommendations in the literature, the centers in this study also perform pulmonary hypertension screening with ECHO at an average of 36 PMA weeks.^[14]^

In patients with sBPD, it is recommended that analgesics and sedatives are avoided, as they are an important risk factor for neurodevelopmental delay.^[11]^ The best indicators of effective ventilation are the patient’s comfort, the absence of ventilator asynchrony without sedation, and healthy growth. Additionally, to achieve the expected benefits from the SIMV-VG + PSV mode in severe BPD with heterogeneous lung parenchyma, the patient’s spontaneous breathing must not be suppressed.^[5]^ In the study, it was observed that sedative analgesics were sometimes used in the BPD patient group and the most frequently used agent was the opioid (Fig. 4C, D).

NIPPV has been shown to reduce extubation failure in several studies and has been reported to be superior to other modes of noninvasive ventilation in this regard.^[3]^ The study found that NIPPV was the most commonly used method of noninvasive ventilation after extubation in patients with sBPD.

At present, the most common indication for tracheostomy is the need for prolonged mechanical ventilation. Therefore, infants with BPD are the most commonly tracheostomised group. However, even in this specific group of patients, the number and timing of tracheostomies varied between centers, as in other studies.^[15]^ This is because the criteria used to decide whether to open a tracheostomy are different in each center, and evidence-based guidelines are lacking. The study found that airway malacia, unsuccessful extubation attempts and clinical instability were the most commonly used criteria for making a tracheostomy decision. The Children’s Hospitals Neonatal Consortium NICU study showed that the most common criteria for tracheostomy placement were airway malacia, ventilation, and pulmonary hypertension, which is consistent with this study.^[16]^ Another study reported that tracheostomy was performed in these patients at 42 to 51 weeks PMA, and this study found that tracheostomy was considered in these patients at an average of 44 weeks PMA, in line with the literature.^[15]^

Although it is difficult to conduct evidence-based studies for BPD, which has a multifactorial etiology and dynamic course, we believe that the use of pathophysiology-based expert recommendations is the most appropriate option for this vulnerable group.

## 6. Conclusion

In the progression from RDS to evolving and severe BPD, changes in the lung under respiratory support require changes in treatment goals and strategies. The lack of evidence-based information in this dynamic process leads to underuse of current recommendations in practice and variation between clinics. Analysis of these differences will provide the basis for prospective and evidence-based studies on this topic. The survey underscores the need for standardized ventilation protocols and improved adherence to evidence-based guidelines in the management of BPD in newborns. It highlights the importance of early identification and intervention, ensuring a smoother transition from acute to chronic ventilation. Additionally, increasing awareness of BPD pathophysiology and optimizing long-term respiratory care strategies. Addressing these gaps will help achieve a more consistent and effective approach to BPD management in the region.

## Author contributions

**Conceptualization:** Funda Tüzün, Nuray Duman, Hasan Özkan.

**Data curation:** Can Akyildiz, Funda Tüzün.

**Formal analysis:** Can Akyildiz.

**Investigation:** Can Akyildiz.

**Methodology:** Nuray Duman.

**Project administration:** Can Akyildiz, Funda Tüzün.

**Supervision:** Nuray Duman, Hasan Özkan.

**Resources:** Funda Tüzün, Abdullah Bariş Akcan, Zeynep Alp Ünkar, Canan Aygün, Şenol Bozdağ, Özlem Bozkurt, Özgül Bulut, Ali Bülbül, Melek Büyükeren, Gökhan Büyükkale, Yalçin Çelik, İstemi Han Çelik, Hasan Çetin, Merih Çetinkaya, Dilek Çoban, Tuğba Egeli, Zeynel Gökmen, Özkan İlhan, Fatih İşleyen, Şebnem Kader, Hasan Kahveci, Gözde Kanmaz, Leyla Karadeniz, Belma Saygili Karagöl, Nejat Narli, Emel Okulu, Hakan Ongun, Mustafa Özdemir, Özmert M.A. Özdemir, Ahmet Özdemir, Hilal Özkan, Hüseyin Şimşek, Sema Tanriverdi, Nuriye Tarakçi, Kadir Şerafettin Tekgündüz, Demet Terek, Özgün Uygur, İpek Güney Varal, Tülin Gökmen Yildirim.

**Writing – original draft:** Can Akyildiz.

**Writing – review & editing:** Can Akyildiz, Funda Tüzün, Nuray Duman, Hasan Özkan.

## Supplementary Material


