# Supplementary material for: Adaptation of ventilation strategies from acute RDS to severe BPD: A national multicenter survey of practices in extremely preterm infants
Source: Medicine (Baltimore). 2025 May 30;104(22):e41973. doi: 10.1097/MD.0000000000041973 (PMC12129518; doi:10.1097/MD.0000000000041973)
Supplement: Supplementary file 1 [file medi-104-e41973-s001.pdf]

Ventilation Strategies from Acute RDS to Severe BPD: A National Multicenter Survey of Practices in Extremely Preterm Infants

1. General Questions about Your Unit

Currently, there is a lack of evidence-based data on the ventilation of infants with BPD. Due to the absence of clear protocols for BPD ventilation, approaches vary from unit to unit. By agreeing to participate in this study, you are expected to answer the questions on behalf of your clinic. The first part of this survey contains information about your unit, and once all data is completed, you will proceed to the second and third parts focusing on ventilation strategies. There are a total of 42 questions, some of which are open-ended. The questions marked with an * are mandatory, and they cannot be skipped without completing them.

Please note that the information obtained from the survey may result in publications or presentations, and the person and institution/hospital system completing the survey will remain anonymous. We appreciate your time, and the survey is expected to take approximately 25-30 minutes.

1. *Email Address:
2. *Name of Your Institution:
3. *Total number of registered level 3 and 4 beds in your unit:
4. *Number of < 28-week infants hospitalised in your NICU **annually:**
5. *Which classification do you use for BPD in your unit?
    a. 2001 NICHD
    b. 2018 updated NICHD
    c. 2019 Neonatal Research Network (Jensen classification)
    d. Other: please specify:
6. *What type of BPD have you seen more frequently in your unit in recent years?
    a. Old BPD
    b. New BPD
    c. Both old and new BPD combined
7. *In the past 3 years, how many severe BPD cases requiring invasive mechanical ventilation at 36 postmenstrual weeks have you had?
8. *In the past 3 years, how many patients have required a tracheostomy due to severe BPD?
9. *In the past 3 years, how many patients have died due to severe BPD?
10. *In the past 3 years, how many patients have been discharged with home oxygen support?

2. Ventilation Strategies during RDS and Progression to BPD

In this section, we would like to ask about your unit's preferences regarding ventilation during the acute phase of RDS and the progression to BPD. The first question concerns how often you use different ventilation modes at various stages. After answering this question, only complete the questions in other areas for the modes you frequently and most frequently use (scored 3 or 4). Skip questions about modes you rarely or never use. Please keep in mind that the patients are extremely preterm infants under 28 weeks.

11. * **During the acute phase of RDS, which mode(s) of invasive mechanical ventilation do you most frequently prefer?** Rate your usage frequency on a scale of 0-4. (**0:** Never | **1:** Very Rare | **2:** Sometimes | **3:** Frequently **|** **4:** Most Frequently) After answering this question, only complete questions 12-15 for the modes you scored 3 or 4. Skip questions about modes you rarely or never use.

**Mode**	**0**	**1**	**2**	**3**	**4**
A/C					
SIMV					
PSV					
A/C-VG					
SIMV-VG					
PSV-VG					
SIMV-VG+PSV					
HFOV					
HFOV-VG					
NAVA					
Other (please specify)					

12. **If you frequently/most frequently use volume-guaranteed synchronized ventilation modes during the acute phase of RDS, indicate the most frequently used values and their minimum-maximum limits for the following parameters.** Skip this question if you do not use these modes frequently.

Parameter	Most Frequent (Min-Max)
Ti (sec)	
VT (ml/kg)	
PEEP (cmH2O)	
Rate (frequency)	
Max PIP (cmH2O)	

13. **If you frequently/most frequently use non-volume-guaranteed synchronized modes during the acute phase of RDS, indicate the most frequently used values and their minimum-maximum ranges for the following parameters.** Skip this question if you do not use these modes frequently.

Parameter	Most Frequent (Min-Max)
Ti (sec)	
PIP (ml/kg)	
PEEP	
Rate (frequency)	

14. If you frequently/most frequently use volume-guaranteed HFOV during the acute phase of RDS, indicate the most frequently used values and their minimum-maximum ranges for the following parameters. Skip this question if you do not use this mode frequently.

Parameter	Most Frequent (Min-Max)
TV hf (ml/kg)	
Frequency (Hz)	
MAP (cmH2O)	
I: E ratio	
Max Amplitude (cmH2O)	

15. If you frequently/most frequently use non-volume-guaranteed HFOV during the acute phase of RDS, indicate the most frequently used values and their minimum-maximum ranges for the following parameters. Skip this question if you do not use this mode frequently.

Parameter	Most Frequent (Min-Max)
Frequency (Hz)	
MAP	
Amplitude (cmH2O)	
I:E ratio	

16. * **Which mode(s) do you most frequently prefer for infants requiring invasive mechanical ventilation for more than 4 weeks, assessed as evolving BPD?** Rate your usage frequency on a scale of 0-4 **(0:** Never | **1:** Very Rare | **2:** Sometimes | **3:** Frequently | **4:** Most Frequently). After answering this question, only complete questions 17-20 for the modes you scored 3 or 4. Skip questions about modes you rarely or never use.

Mode	0	1	2	3	4
A/C					
SIMV					
PSV					
A/C-VG					
SIMV-VG					
PSV-VG					
SIMV-VG+PSV					
HFOV					
HFOV-VG					
NAVA					
Other (please specify)					

17. **If you frequently/most frequently use volume-guaranteed conventional modes for infants requiring invasive mechanical ventilation for more than 4 weeks, assessed as evolving BPD, indicate the most frequently used values and their minimum-maximum ranges for the following parameters.** Skip this question if you do not use these modes frequently.

Parameter	Most Frequent (Min-Max)
Ti (sec)	
VT (ml/kg)	
PEEP	
Rate (frequency)	
Max PIP (cmH2O)	

18. **If you frequently/most frequently use non-volume-guaranteed synchronized modes for infants requiring invasive mechanical ventilation for more than 4 weeks, assessed as progressing to BPD, indicate the most frequently used values and their minimum-maximum ranges for the following parameters.** Skip this question if you do not use these modes frequently.

Parameter	Most Frequent (Min-Max)
Ti (sec)	
PIP (ml/kg)	
PEEP (cmH2O)	
Rate (frequency)	
I:E ratio	

19. If you frequently/most frequently use **volume-guaranteed HFOV for infants requiring invasive mechanical ventilation for more than 4 weeks, assessed as evolving BPD**, indicate the most frequently used values and their minimum-maximum ranges for the following parameters. Skip this question if you do not use this mode frequently.

Parameter	Most Frequent (Min-Max)
TV hf (ml/kg)	
Frequency (Hz)	
MAP (cmH2O)	
I:E ratio	
Max Amplitude (cmH2O)	

20. If you frequently/most frequently use **non-volume-guaranteed HFOV** for infants requiring invasive mechanical ventilation for more than 4 weeks, assessed as progressing to BPD, indicate

the most frequently used values and their minimum-maximum ranges for the following parameters. Skip this question if you do not use this mode frequently.

**Parameter**	**Most Frequent (Min-Max)**
Frequency (Hz)	
MAP	
Amplitude (cmH2O)	
I:E ratio	

3. Ventilation Strategies in Severe BPD

In this section, we ask about the mechanical ventilation strategies and approaches of your unit for infants with severe BPD (Grade 3) requiring invasive mechanical ventilation at 36 postmenstrual weeks.

21. *Which ventilation mode(s) do you most frequently prefer for infants requiring invasive mechanical ventilation, assessed as severe BPD? Rate your usage frequency on a scale of 0-4. After answering this question, only complete questions 22-25 for the modes you scored 3 or 4. Skip questions about modes you rarely or never use.

0: Never | 1: Very Rare | 2: Sometimes | 3: Frequently | 4: Most Frequently

**Mode**	**0**	**1**	**2**	**3**	**4**
A/C					
SIMV					
PSV					
A/C-VG					
SIMV-VG					
PSV-VG					
SIMV-VG+PSV					
HFOV					
HFOV-VG					
NAVA					
Other (please specify)					

22. If you frequently/most frequently use **volume-guaranteed conventional modes** for infants with **severe BPD** requiring invasive mechanical ventilation, indicate the most frequently used values and their minimum-maximum ranges for the following parameters. Skip this question if you do not use these modes frequently.

**Parameter**	**Most Frequent (Min-Max)**
Ti (sec)	
VT (ml/kg)	
PEEP	
Max PIP (cmH2O)	
Rate (frequency)	
I:E ratio	

23. If you frequently/most frequently use **non-volume-guaranteed synchronized modes for infants with severe BPD** requiring invasive mechanical ventilation, indicate the most frequently used values and their minimum-maximum ranges for the following parameters. Skip this question if you do not use these modes frequently.

**Parameter**	**Most Frequent (Min-Max)**
Ti (sec)	
PIP (ml/kg)	
PEEP (cmH2O)	
Rate (frequency)	
I:E ratio	

24. If you frequently/most frequently use **volume-guaranteed HFOV** for infants with severe BPD requiring invasive mechanical ventilation, indicate the most frequently used values and their minimum-maximum ranges for the following parameters. Skip this question if you do not use this mode frequently.

**Parameter**	**Most Frequent (Min-Max)**
TV hf (ml/kg)	
Frequency (Hz)	

Parameter	Most Frequent (Min-Max)
MAP (cmH2O)	
I:E ratio	
Max Amplitude (cmH2O)	

25. If you frequently/most frequently use non-volume-guaranteed HFOV for infants with severe BPD requiring invasive mechanical ventilation, indicate the most frequently used values and their minimum-maximum ranges for the following parameters. Skip this question if you do not use this mode frequently.

Parameter	Most Frequent (Min-Max)
Frequency (Hz)	
MAP	
Amplitude (cmH2O)	
I:E ratio	

26. *What parameters do you consider when adjusting tidal volume, inspiratory time, and respiratory rate in severe BPD? Rank them in order of importance.

Insignificant | Slightly Important | Important | Very Important | Most Important

Parameter	Insignificant	Slightly Important	Important	Very Important	Most Important
Heterogeneous parenchymal appearance					
Appearance of overinflation					
CO2 retention					
Oxygenation					
Extent of atelectasis					
Respiratory rate of the patient					

Please specify other parameters you consider very important:

27. *What is your target pCO_2 range (mmHg) for infants with severe BPD requiring invasive mechanical ventilation? Specify Min-Max values.

28. *How do you most frequently monitor pCO_2 in infants with severe BPD requiring invasive mechanical ventilation?

    a. Blood gas

    b. Transcutaneous CO_2 monitor

    c. End-tidal CO_2 monitor

29. *What is your target arterial oxygen saturation (% SpO_2) range for infants with severe BPD requiring invasive mechanical ventilation? Specify Min-Max values.

30. *Rate your preference for using sedative and/or analgesic agents during invasive mechanical ventilation in infants with severe BPD, based on usage frequency.

Never | Very Rarely | Sometimes | Most of the Time | Always

**Mode**	**Never**	**Very Rarely**	**Sometimes**	**Most of the Time**	**Always**
Sedative/Analgesic					
Other (please specify)					

If you use sedative and/or analgesic agents most of the time, specify the agent you use most frequently:

31. *At what FiO_2 level do you plan to extubate if other parameters are appropriate for extubation in an infant with severe BPD?

    a. < 30%

    b. < 40%

    c. < 50%

    d. < 60%

    e. Other (please specify):

32. *Do you routinely plan echocardiography screening for pulmonary hypertension in cases of advanced BPD?

    - Yes (Please specify the time) :
    - No

33. * Which mode(s) do you most frequently prefer when transitioning from invasive to non-invasive respiratory support in infants with severe BPD? Rate your usage frequency on a scale of 0-4.

0: Never | 1: Very Rare | 2: Sometimes | 3: Frequently | 4: Most Frequently

**Mode**	**0**	**1**	**2**	**3**	**4**
NAVA					
Synchronized NIPPV					
NIPPV					
nHFOV					
CPAP					
HFNC					
Other (please specify)					

34. If you frequently/most frequently use NIPPV during the transition from invasive to non-invasive mode in infants with severe BPD, indicate the most frequently used values and their minimum-maximum ranges for the following parameters. Skip this question if you do not use this mode frequently.

**Parameter**	**Most Frequent (Min-Max)**
PEEP	
PIP	
Rate (Frequency)	
Ti	

35. If you frequently/most frequently use CPAP during the transition from invasive to non-invasive mode in infants with severe BPD, indicate the most frequently used PEEP values and their minimum-maximum ranges for the following parameters. Skip this question if you do not use this mode frequently.
    Most Frequent: ………………. (Min-Max): …………………….

36. If you frequently/most frequently use nasal HFOV during the transition from invasive to non-invasive mode in infants with severe BPD, indicate the most frequently used values and their minimum-maximum ranges for the following parameters. Skip this question if you do not use this mode frequently.

Parameter	Most Frequent (Min-Max)
Frequency (Hz)	
MAP	
Amplitude (cmH2O)	
I:E ratio	

37. *What is the highest pCO2 pressure (mmHg) you tolerate in an infant with severe BPD who has transitioned to non-invasive mode to avoid reintubation?

    ……………………….

38. *What is the highest FiO2 (%) you tolerate in an infant with severe BPD who has transitioned to non-invasive mode to avoid reintubation?

    ………………………….

39. *What is the **maximum number of systemic postnatal steroid therapy courses (minimum 5 days)** you apply in infants with BPD who cannot be weaned from mechanical ventilation?

    …………………………….

40. *Rate the parameters you consider when contemplating tracheostomy in infants with severe BPD according to the importance given to these indicators in your institution.

Insignificant | Slightly Important | Important | Very Important | Most Important

**Parameter**	**Insignificant**	**Slightly Important**	**Important**	**Very Important**	**Most Important**
Multiple failed extubation attempts after 36 postmenstrual weeks					
Growth restriction					
Airway malacia					
Physiological instability					
Inability to extubate despite repeated steroid courses					
Recurrent sepsis attacks					
Presence of pulmonary hypertension					

**Parameter**	**Insignificant**	**Slightly Important**	**Important**	**Very Important**	**Most Important**
Exceeding a certain postconceptional week					
Exceeding a certain postnatal day					
Extreme agitation and restlessness					
Family decision and social status					

Please specify other very important reasons:

41. *Is there a specific postmenstrual week at which you strongly consider performing a tracheostomy if an infant with severe BPD cannot be extubated?

……………………….

42. *Is there a specific postnatal day at which you strongly consider performing a tracheostomy if an infant with severe BPD cannot be extubated?

……………………….
